# Perioperative Blood Biomarkers of Infectious and Non-Infectious Postoperative Pulmonary Complications: A Narrative Review

**DOI:** 10.3390/jcm15020699

**Published:** 2026-01-15

**Authors:** Simona Gigliotti, Giuseppe Guerriero, Giuseppe Mazza, Eugenio Garofalo, Grazia Pavia, Angela Amaddeo, Antonia Rizzuto, Nadia Marascio, Angela Quirino, Federico Longhini, Giovanni Matera

**Affiliations:** 1 Department of Health Sciences, “Magna Græcia” University, 88100 Catanzaro, Italy; graziapavia@unicz.it (G.P.); nmarascio@unicz.it (N.M.); quirino@unicz.it (A.Q.); mmatera@unicz.it (G.M.); 2 Unit of Clinical Microbiology, “R. Dulbecco” Teaching Hospital, 88100 Catanzaro, Italy; 3 Department of Medical and Surgical Sciences, “Magna Græcia” University, 88100 Catanzaro, Italy; giu.guerriero@outlook.it (G.G.); mazzagiu@gmail.com (G.M.); eugenio.garofalo@unicz.it (E.G.); angela.amaddeo@gmail.com (A.A.); arizzuto@unicz.ii (A.R.); flonghini@unicz.it (F.L.); 4 Anesthesia and Intensive Care Department, “R. Dulbecco” Teaching Hospital, 88100 Catanzaro, Italy; 5 General Surgery Unit, “R. Dulbecco” Teaching Hospital, 88100 Catanzaro, Italy

**Keywords:** postoperative pulmonary complications, postoperative pulmonary infection, blood biomarkers, c-reactive protein, procalcitonin, inflammation, interleukin, cytokine, tumor necrosis factor-alpha, oxidative stress, endothelial dysfunction

## Abstract

**Background/Objectives:** Postoperative pulmonary complications (PPCs) remain frequent and increase morbidity, mortality, and resource use. Preoperative risk scores (ARISCAT, NSQIP-derived calculators) use mostly static variables and may miss the dynamic perioperative host response preceding respiratory deterioration or infection. We address the gap in clinically interpretable syntheses of perioperative blood biomarker trajectories that distinguish infectious from non-infectious PPCs and clarify bedside-ready versus exploratory markers. **Methods:** We conducted a narrative review with a structured Medline search (inception to 1 November 2025) plus reference screening. We included English-language adult surgical studies (observational or interventional) evaluating perioperative blood biomarkers in relation to PPCs or postoperative pulmonary infection; case reports, editorials, and reviews were excluded. No formal risk-of-bias assessment or quantitative meta-analysis was performed. **Results:** Across 298 cited publications, serial patterns of routinely available biomarkers (C-reactive protein, procalcitonin, lactate, albumin, and leukocyte-derived indices) were most consistently associated with PPC risk and helped separate expected postoperative inflammation from evolving infection when interpreted longitudinally rather than as single values. Mechanistic biomarkers (cytokines/immune-function assays, endothelial injury and coagulation/fibrinolysis markers, oxidative stress indicators) add biological insight but are limited by assay availability, heterogeneous sampling windows, and absent standardized cut-offs. Omics signatures and machine learning models combining biomarker kinetics with clinical variables are promising but require prospective, transportable validation. **Conclusions:** Key barriers to implementation include biological variability, non-specificity across postoperative syndromes, heterogeneous sampling windows, and lack of standardized cut-offs. Integrating multimarker panels into validated, dynamic predictive frameworks represents a promising direction for perioperative precision medicine.

## 1. Introduction

Postoperative pulmonary complications (PPCs) remain among the most persistent and clinically consequential challenges in contemporary perioperative medicine [1,2]. Despite advances in surgical techniques, anaesthetic monitoring, intensive care practice, and enhanced recovery protocols [3,4,5,6,7,8], PPCs remain common across surgical populations and are consistently associated with prolonged hospitalisation, increased ICU utilisation, higher healthcare costs, reduced postoperative quality of life, and increased morbidity and mortality [9,10]. This burden is further amplified by pulmonary infections caused by multidrug-resistant (MDR) organisms [11].

PPCs are not a single entity but an umbrella term encompassing a heterogeneous set of respiratory disturbances with distinct mechanisms and prognostic implications, including postoperative pneumonia, atelectasis, bronchospasm, respiratory failure requiring non-invasive or invasive ventilatory support, pulmonary oedema, aspiration events, and, in severe cases, acute respiratory distress syndrome (ARDS) [12]. Although clinicians routinely apply perioperative risk assessment tools, commonly used models such as ARISCAT and ACS-NSQIP rely predominantly on static preoperative variables and may not adequately capture the dynamic biological responses unfolding during and after surgery [13,14].

This mismatch between clinical risk stratification and evolving perioperative physiology highlights the need for biomarkers, objective, quantifiable indicators of biological processes at molecular, cellular, and systemic levels [15]. Blood biomarkers are particularly attractive because they can be measured serially, interpreted rapidly, and incorporated into both conventional and machine-learning-based prediction models. Importantly, PPCs often develop along a continuum, preceded by measurable perturbations in inflammation, immune competence, endothelial integrity, oxidative balance, and metabolic stability [12,16].

Surgical trauma triggers coordinated systemic responses, including inflammatory activation, stress signalling, immune-cell redistribution, endothelial dysfunction, coagulation/fibrinolysis imbalance, and metabolic derangements [17,18,19,20,21,22], that directly intersect with pulmonary vulnerability: inflammation may increase endothelial permeability [23], perioperative immune suppression may increase susceptibility to bacterial pneumonia [24,25], oxidative stress may predispose to ventilator-associated lung injury [26,27,28], and procoagulant shifts may promote microthrombi and ventilation-perfusion mismatch [29,30]. Accordingly, blood biomarkers provide a pragmatic window into mechanisms that may precede clinical deterioration.

Over the past decade, studies have evaluated numerous candidate biomarkers, ranging from routinely available markers (e.g., CRP, PCT, leukocyte-derived indices, albumin) to less accessible assays reflecting specific pathways (e.g., cytokine signalling, endothelial injury, oxidative stress, and coagulation activation) [31,32,33,34]. Emerging “omics” approaches (including circulating microRNAs, proteomic signatures, and metabolomic profiles) may further refine perioperative risk phenotyping, but most remain investigational [35,36,37,38,39].

Despite this progress, translation into routine practice remains limited by inter-individual variability, lack of standardised cut-offs, uncertainty regarding optimal sampling windows, and difficulties distinguishing pulmonary-specific complications from systemic inflammatory or infectious processes [40,41,42,43,44,45]. Nevertheless, biomarker-guided prediction and monitoring is gaining momentum and may support decision-making across antimicrobial initiation, ventilatory strategies, fluid management, triage, and postoperative surveillance intensity [46,47,48].

In this narrative review, we synthesise perioperative blood biomarkers associated with infectious and non-infectious PPCs and organise them into physiologically meaningful domains and perioperative trajectories to support clinically interpretable phenotyping. We also discuss current limitations and future directions, including integration into multimodal predictive frameworks and the potential contribution of omics and machine learning to perioperative precision medicine.

## 2. Materials and Methods

This article is a narrative review; therefore, it does not aim to provide an exhaustive systematic evidence synthesis or a quantitative meta-analysis. To inform the narrative synthesis, we performed a structured literature search in Medline (via PubMed) from inception to 1 November 2025 using combinations of terms related to PPCs and biomarkers. Blocks of terms per concept have been created [49]. Two authors (SG and GG) independently executed the search using keywords and their related MeSH terms such as: “pulmonary complications”, “postoperative pulmonary complications”, “postoperative sepsis”, “postoperative infection”, “biomarkers”, “inflammation mediators”, “c-reactive protein”, “procalcitonin”, “inflammation”, “interleukin”, “cytokine”, “tumor necrosis factor-alpha”, “HLA-DR”, “neutrophils”, “lymphocytes”, “malondialdehyde”, “oxidative stress”, “endothelial dysfunction”. The constructed search strategy is reported in the Supplementary Materials.

We included English-language studies in adults (>18 years) undergoing any type of surgery that evaluated perioperative blood biomarkers (pre-, intra-, or postoperative; serum/plasma/whole blood) in relation to PPCs or postoperative pulmonary infection. Eligible designs comprised observational studies (prospective/retrospective cohorts, case–control, or cross-sectional analyses) and interventional studies/trials reporting biomarker associations or performance. Excluded were case reports, review articles, editorials, and studies available solely in abstract form [49]. To identify any overlooked studies of relevance, we examined the references of included papers. Titles and abstracts have been independently screened by two authors (SG and GG) according to the inclusion criteria and the full texts of the potentially relevant reports have been retrieved and examined. Any discrepancies were resolved through consultation with a third examiner (FL) [49].

We extracted and narratively synthesized biomarker patterns, emphasizing longitudinal trajectories and mechanistic domains. No protocol was registered, no formal risk-of-bias assessment was performed, and no quantitative pooling was attempted. A study selection flow diagram is provided as Supplementary Material (Figure S1) for transparency [49].

## 3. Definition and Microbiology of Postoperative Pulmonary Complications

In broad clinical usage, PPC is an umbrella term that encompasses “almost any” respiratory-system complication occurring after anaesthesia and surgery [12,50]. However, historically, PPC research has been limited by heterogeneous endpoints and inconsistent operational definitions, which complicates benchmarking across studies and undermines comparability of preventive and therapeutic trials [51].

To address this, consensus groups have proposed standardised definitions. Two widely cited frameworks are the European Perioperative Clinical Outcome (EPCO) definitions, a broad composite PPC outcome intended for perioperative trials and quality assessment [52], and the Standardised Endpoints in Perioperative Medicine-Core Outcome Measures in Perioperative and Anaesthetic Care (StEP-COMPAC) definitions, a mechanism-focused composite PPC outcome designed to improve biological coherence and include an explicit severity assessment [51].

The EPCO framework takes a broader clinical approach and groups several postoperative pulmonary events under PPCs, including respiratory infection, respiratory failure, pleural effusion, atelectasis, pneumothorax, bronchospasm, and aspiration pneumonitis [52]. EPCO also treats several major pulmonary diagnoses (i.e., ARDS, pneumonia, pulmonary embolism) as important perioperative outcomes that can be reported as separate endpoints depending on study aims [52]. On the opposite, StEP-COMPAC defines PPCs as a composite of respiratory diagnoses that share common mechanisms (predominantly lung collapse and/or airway contamination). The composite includes atelectasis, pneumonia, pulmonary aspiration, and ARDS [51]. From a biomarker perspective, standardized PPC definitions are essential because endpoint heterogeneity (infectious vs. non-infectious events and severity grading) directly affects the inflammatory signal being measured and, therefore, the interpretability and comparability of biomarker trajectories.

Within the spectrum of PPCs, infectious events, particularly postoperative pneumonia and postoperative tracheobronchitis, are among the most clinically consequential because they can rapidly progress to respiratory failure, prolong hospitalisation, and drive ICU utilisation.

Pathophysiologically, perioperative factors (general anaesthesia, intubation, impaired cough and mucociliary clearance, atelectasis, pain-related hypoventilation, aspiration risk, and exposure to healthcare flora) create a “perfect storm” for airway contamination and impaired clearance, facilitating bacterial overgrowth and infection, particularly when postoperative ventilation or critical care is required [53,54,55,56]. Contemporary cohorts of severe postoperative respiratory infections show that these events often occur early after surgery and are frequently nosocomial in nature, with substantial rates of Gram-negative and MDR isolates [11,57]. Across surgical populations, the microbiology of postoperative pneumonia is commonly dominated by aerobic Gram-negative bacilli and Staphylococcus aureus (including MRSA in higher-risk settings), although distributions vary by procedure, baseline patient risk, and care environment (ward vs. ICU). Reviews and observational data repeatedly identify Enterobacterales (i.e., *Klebsiella*, *Enterobacter* spp.), Pseudomonas aeruginosa, and S. aureus as frequent pathogens [55,58]. In severe postoperative respiratory infections requiring ICU admission, microbiological sampling is typically high and yields a substantial proportion of isolates; importantly, these cohorts also report notable MDR rates. In one prospective ICU cohort of severe postoperative pneumonia/tracheobronchitis, diagnosis occurred a mean of ~6 days after surgery and isolates included a high proportion of Gram-negative and MDR bacteria (~20%) [11]. Other surgical series similarly report meaningful MDR proportions in postoperative pneumonia (~17% in one case–control study), reinforcing the need for structured risk stratification and empiric-therapy choices guided by local ecology [59,60].

Postoperative sepsis is among the most severe postoperative infectious syndromes and a major contributor to mortality after surgery [61]. Postoperative pneumonia is a frequent infectious source in surgical patients and can precipitate systemic deterioration [62]. Sepsis is also a leading precipitant of ARDS, and the combination of septic shock and ARDS is associated with markedly worse outcomes [63]. These severe trajectories underscore the need for early stratification and timely diagnostic decisions, an area where perioperative biomarker kinetics may support earlier recognition of evolving infection and impending respiratory failure [63,64,65,66,67,68].

## 4. Pathophysiology of Postoperative Pulmonary Complications

The pathophysiology of PPCs is multifaceted, reflecting interactions between systemic biological responses, local pulmonary processes, mechanical ventilation effects, and patient-level risk factors. Understanding these mechanisms in depth is essential for interpreting biomarkers, as each biomarker reflects a particular component of the biological cascade that contributes to PPCs. This is consistent with biomarker-driven personalization frameworks for sepsis risk that integrate perioperative inflammation, immune dysregulation, and endothelial injury to explain heterogeneous postoperative infectious trajectories [69].

### 4.1. Systemic Inflammation and the Surgical Stress Response

Surgical trauma, regardless of the anatomical site, initiates an immediate systemic inflammatory response. Tissue injury activates pattern recognition receptors on macrophages, neutrophils, and dendritic cells, leading to release of cytokines such as interleukin-6 (IL-6), interleukin-8 (IL-8), tumour necrosis factor-α (TNF-α), and various chemokines [70].

Systemic inflammation results in widespread endothelial activation, increased capillary permeability [71], and recruitment of immune cells to distant organs, including the lungs [72]. Pro-inflammatory cytokines can disrupt the pulmonary microvascular endothelial barrier, increasing alveolar-capillary permeability and enabling leakage of protein-rich fluid into the interstitium and alveolar spaces, an early hallmark of non-cardiogenic pulmonary oedema and ARDS [73,74,75,76]. Simultaneously, activated neutrophils release reactive oxygen species (ROS) and proteolytic enzymes (notably neutrophil elastase and matrix metalloproteinases) that damage endothelial and epithelial junctions, thereby worsening alveolar-capillary barrier dysfunction and promoting permeability oedema and lung injury [77,78,79,80].

Although inflammation is essential for tissue healing [81,82,83], excessive or dysregulated inflammation can push the lungs toward injury [84,85]. The delicate structure of the pulmonary microvasculature makes it particularly vulnerable. As the systemic inflammatory load increases, the balance between pro-inflammatory and anti-inflammatory pathways shifts unfavourably, setting the stage for respiratory dysfunction [23,73,86].

Systemic inflammation also increases metabolic demands. In the postoperative period, this higher oxygen requirement, combined with compromised ventilation and gas exchange (e.g., pain-limited inspiratory effort, residual sedatives/opioids, and perioperative atelectasis), can reduce physiologic reserve and accelerate progression toward respiratory insufficiency [21,87,88,89]. Biomarkers such as CRP, IL-6, IL-8, and TNF-α provide insight into the magnitude of systemic inflammation and help identify patients at risk of pulmonary deterioration [90,91,92,93].

### 4.2. Immune Dysregulation and Postoperative Immune Suppression

Following the initial postoperative pro-inflammatory surge, the immune system often shifts toward a compensatory anti-inflammatory response (CARS), intended to limit collateral tissue damage but potentially resulting in clinically relevant immunosuppression [22,94,95]. This phase is commonly characterized by downregulated monocyte HLA-DR expression (reflecting reduced antigen-presenting capacity), postoperative lymphopenia, and functional defects in innate immunity, such as impaired neutrophil migration/chemotaxis, which collectively increase susceptibility to secondary infections, including postoperative pneumonia [96,97,98,99].

This postoperative immunologic “downregulation” increases susceptibility to postoperative pneumonia, one of the most frequent and clinically consequential PPCs [12,50,100]. Under normal conditions, inhaled pathogens are contained by airway clearance mechanisms and eliminated by resident alveolar macrophages, with rapid recruitment of neutrophils when needed [101]. After major surgery, however, pulmonary and systemic innate defences can be impaired: reduced HLA-DR expression is a marker of diminished antigen presentation, while postoperative lymphopenia weakens adaptive immune responses, allowing bacteria that would otherwise be cleared to proliferate and cause infection [96,97,102,103]. Residual sedation/opioids, impaired cough, perioperative atelectasis, microaspiration, and reduced mucociliary transport can further compound this vulnerability [12,103,104].

Biomarkers reflecting immune suppression, including low monocyte HLA-DR expression, elevated neutrophil-to-lymphocyte ratio (NLR), and shifts in immune-cell subsets, may help identify patients entering a state of heightened immunologic vulnerability [96,97,105]. Finally, the interplay between inflammation and immune suppression is increasingly recognized as dynamic and bidirectional: a pronounced early inflammatory response can be followed by compensatory immune dysfunction, and both phases may contribute to PPC risk [106,107,108].

### 4.3. Endothelial Dysfunction and Vascular Permeability

The pulmonary endothelium is central to efficient gas exchange, regulation of fluid homeostasis, and control of leukocyte trafficking across the alveolar-capillary interface. Surgical stress can provoke endothelial injury through multiple, often converging mechanisms, including inflammatory cytokine signalling, oxidative stress, and iatrogenic mechanical forces during positive-pressure ventilation [23,73,109,110].

Endothelial dysfunction is typically characterized by disruption of inter-endothelial junctions and increased alveolar-capillary permeability, alongside endothelial activation with upregulation and shedding of adhesion molecules (e.g., ICAM-1 and VCAM-1), which promotes leukocyte adhesion and transmigration into lung tissue. In parallel, oxidative stress can reduce nitric oxide (NO) bioavailability, impairing vasodilation and worsening microvascular dysregulation [23,73,111].

As permeability increases, protein-rich fluid shifts from the intravascular space into the interstitium and alveoli, manifesting clinically as pulmonary oedema and contributing to impaired oxygenation. Endothelial injury is therefore a key pathophysiological step in acute lung injury and progression to ARDS [73,112,113].

Circulating biomarkers, including soluble ICAM-1, soluble VCAM-1, von Willebrand factor (vWF), and angiopoietins (particularly angiopoietin-2 and the Ang-2/Ang-1 balance), provide insight into endothelial activation and barrier disruption. Elevated levels reflect systemic vascular stress and have been linked to pulmonary vascular dysfunction and adverse respiratory outcomes, including in perioperative settings [45,112,113,114].

Endothelial activation can be especially pronounced after cardiopulmonary bypass, major trauma, or prolonged mechanical ventilation. In these high-risk contexts, tracking endothelial biomarkers, particularly perioperative Ang-2 and markers of endothelial activation, may help anticipate postoperative hypoxaemia and support more individualized decisions regarding fluid strategy, ventilatory settings, and monitoring intensity [45,114,115,116].

### 4.4. Oxidative Stress and Mitochondrial Dysfunction

Oxidative stress arises when the generation of ROS exceeds the capacity of endogenous antioxidant defences. In the perioperative setting, surgery can promote oxidative stress through several converging mechanisms, including ischemia-reperfusion during vascular manipulation, exposure to high inspired oxygen fractions (hyperoxia) during mechanical ventilation, activation of neutrophils with a respiratory burst, and inflammation-related mitochondrial dysfunction [117,118,119,120].

In the lungs, oxidative stress can injure alveolar epithelial cells, impair surfactant homeostasis, and amplify endothelial dysfunction, thereby worsening ventilation-perfusion matching and increasing alveolar-capillary permeability. Experimental and translational studies show that mechanical ventilation, particularly when combined with hyperoxia, can trigger oxidative injury and reduce surfactant-associated proteins, contributing to oedema and impaired gas exchange [26,121,122].

Mitochondrial dysfunction may further exacerbate postoperative respiratory instability. When mitochondrial ATP production is impaired, respiratory (and peripheral) muscle performance can decline, limiting a patient’s capacity to sustain effective ventilation, particularly in patients who require prolonged ventilatory support or develop critical illness-associated weakness [123,124,125,126]. Biomarkers reflecting mitochondrial stress (i.e., circulating mtDNA and other metabolic/oxidative signatures) are still emerging, but systematic reviews suggest they may eventually help identify patients at increased risk of adverse respiratory outcomes [127].

### 4.5. Coagulation Abnormalities and Microthrombosis

Systemic inflammation activates the coagulation cascade and promotes a hypercoagulable, thrombo-inflammatory state. Pro-inflammatory mediators induce tissue factor-driven thrombin generation and are accompanied by reduced activity of endogenous anticoagulant pathways and suppressed fibrinolysis, favouring fibrin deposition and microthrombus formation [128,129,130]. In the pulmonary microcirculation, microthrombosis and dysregulated perfusion can contribute to ventilation-perfusion mismatch (and increased physiologic dead space), thereby worsening hypoxaemia even when ventilation appears adequate [131,132,133].

Markers such as D-dimer, fibrinogen, platelet activation indices, and thrombin-antithrombin (TAT) complexes provide measurable evidence of coagulation activation. D-dimer reflects fibrin formation and subsequent fibrinolysis, but it is nonspecific and commonly rises after surgery and in many inflammatory conditions; interpretation therefore requires clinical context and awareness of its postoperative kinetics [134,135].

Nevertheless, a markedly elevated or rising D-dimer (together with other coagulation markers) may indicate heightened thrombo-inflammatory activation and can help identify patients at increased risk of thrombotic pulmonary complications, particularly pulmonary embolism [134,136,137].

Coagulation responses are especially pronounced in settings with high baseline thrombotic risk, most notably major orthopaedic procedures (hip/knee arthroplasty) and cancer surgery, and can be further amplified by immobility and systemic inflammation, increasing the likelihood of pulmonary embolism or microvascular thrombotic injury [138,139,140]. Recent evidence further supports the value of dynamic hematological indices for early sepsis risk stratification, including perioperative changes in NLR and platelet patterns/PLR, reinforcing derived ratios as accessible biomarkers of systemic immunothrombotic responses relevant to postoperative infectious trajectories [141].

### 4.6. Atelectasis, Impaired Lung Mechanics, and Ventilator Interactions

While circulating biomarkers capture systemic risk, local, lung-specific factors also play a major role in the development of PPCs. Perioperative atelectasis develops rapidly during general anaesthesia, largely due to reductions in functional residual capacity with supine positioning and loss of respiratory muscle tone/diaphragmatic displacement, together with perioperative impairment of surfactant function that favours alveolar collapse [12,142,143].

Collapsed lung regions contribute to shunt and impaired gas exchange and may also promote local biological vulnerability, including immune dysregulation and increased susceptibility to infection [142]. In addition, mechanical ventilation of a heterogeneously aerated lung can concentrate stress at interfaces between aerated and collapsed units. When atelectatic areas cyclically reopen, higher opening pressures and shear forces may be required, and excessive or repetitive recruitment-derecruitment can aggravate epithelial and endothelial injury (“atelectrauma”) [144,145,146].

Mechanical ventilation can itself contribute to lung injury if not carefully managed. Excessive tidal volumes, insufficient positive end-expiratory pressure, and/or high driving pressures can precipitate ventilator-induced lung injury through barotrauma, volutrauma, atelectrauma (repetitive recruitment-derecruitment), and biotrauma [4,147,148,149,150]. Biotrauma refers to the inflammatory response induced by injurious mechanical stress, with increased pulmonary and systemic release of mediators (i.e., IL-6, IL-8, TNF-α) that can amplify endothelial dysfunction, increase alveolar-capillary permeability, and worsen oedema and hypoxemia [148,151,152].

In both ARDS and the intraoperative setting, clinical and translational evidence supports the importance of limiting mechanical stress, particularly by reducing tidal volume and avoiding excessive driving pressure, to mitigate downstream injury and postoperative pulmonary complications [153,154,155,156,157].

Although ventilator mechanics are not directly captured by blood biomarkers, systemic inflammatory markers can reflect the biological consequences of ventilation-induced stress and may help signal patients in whom mechanical ventilation is contributing to a deleterious inflammatory trajectory [148,152].

### 4.7. Metabolic Stress and Respiratory Muscle Fatigue

Surgery induces a hypermetabolic stress response: increases in cortisol, catecholamines, and inflammatory mediators raise resting energy expenditure and whole-body oxygen demand [21,158]. When respiratory mechanics are compromised by pain-limited inspiratory effort, residual sedation or opioid effects, or perioperative neuromuscular/diaphragmatic weakness, the resulting mismatch between ventilatory workload and muscle capacity becomes clinically relevant. Progressive respiratory muscle fatigue can then lead to hypoventilation, hypercapnia, and, in susceptible patients, postoperative respiratory failure [159,160,161].

In this context, lactate is a useful integrative biomarker; elevated levels may reflect inadequate oxygen delivery relative to demand and/or increased anaerobic glycolysis (while acknowledging that hyperlactatemia can also occur via non-hypoxic mechanisms in stress states) [162,163].

Markers of nutritional reserve such as albumin (and, in some settings, prealbumin) are commonly used to capture frailty, inflammation, and nutritional risk; perioperative hypoalbuminemia and low prealbumin have been associated with worse postoperative outcomes, including pulmonary complications, plausibly through reduced physiologic reserve and impaired muscle performance [164,165]. Patients with poor baseline nutrition, cachexia, or sarcopenia are therefore at heightened risk of postoperative respiratory muscle insufficiency and pulmonary complications [166,167].

## 5. Current Clinical Predictors and Their Limitations

### 5.1. Clinical Risk Scores

For decades, perioperative medicine has relied on clinical risk scores as the main strategy for stratifying a patient’s likelihood of developing PPCs. Tools such as ARISCAT, NSQIP-derived pulmonary risk calculators for pneumonia or postoperative respiratory failure, and procedure- or specialty-specific indices were designed to combine patient characteristics, comorbidities, and operative factors into a single numerical estimate of risk [14,168,169,170,171].

These models typically prioritize variables available before surgery, including age, smoking status, baseline oxygen saturation, chronic respiratory disease, functional status, emergency status, surgical site/type, and anticipated procedure duration, making them attractive because they are simple, widely accessible, and easy to communicate during preoperative counselling [14,168,169].

However, it is increasingly evident that, even when historically useful, these tools have inherent limitations in contemporary perioperative practice. Their fundamentally static structure means they cannot capture the dynamic physiological processes that evolve intraoperatively and in the early postoperative period [47,172].

A patient categorized as low-risk based on preoperative variables may still experience profound inflammatory activation, ventilatory stress, hemodynamic instability, or postoperative immune dysfunction, all of which can sharply increase PPC risk but lie outside the scope of most preoperative scores [12,47,172].

Moreover, many risk scores generalize risk using population-level associations rather than identifying the biological pathways that predispose an individual to pulmonary deterioration. As a result, two patients with similar scores may follow very different trajectories, for example, one progressing toward hyperinflammation while another develops compensatory immune suppression, yet clinical scores are largely “phenotype-blind” to these mechanistic differences and therefore cannot readily guide targeted prevention or treatment [47,173].

Finally, the performance of these models can vary across hospitals, surgical specialties, and patient populations, reflecting differences in case-mix, perioperative pathways, anaesthetic practices, and definitions of PPCs [173,174]. Even when discrimination is acceptable, these tools often provide limited insight into why risk exists or how it changes over time. Together, these limitations support the need for biological markers that can enrich, refine, or, in specific contexts, potentially outperform purely clinical prediction systems [47,173,174].

### 5.2. Blood Biomarkers in Perioperative Care

The limited ability of purely clinical and physiological predictors to detect early biological vulnerability highlights the need for blood biomarkers that can track evolving pathophysiological states across the perioperative timeline. Biomarkers provide a dynamic and quantifiable view of the host response to surgical injury, information that even well-validated preoperative risk models cannot fully capture because perioperative biology changes rapidly during and after the operation [47,172,173].

Surgical trauma initiates a coordinated molecular cascade: cytokine signalling intensifies, immune-cell distributions and functions shift, endothelial activation develops, oxidative defences may be overwhelmed, and coagulation pathways engage. These processes can unfold hours to days before pulmonary symptoms become clinically apparent [22,175,176].

Because biomarkers are directly linked to these mechanisms, they are well positioned to reveal “invisible” transitions, such as escalating systemic inflammation or emerging immune dysfunction, before overt respiratory deterioration [97,176].

Importantly, biomarkers offer mechanistic clarity in addition to prediction. Whereas traditional risk scores primarily indicate who is at risk, biomarker patterns can help suggest why risk exists, whether driven by hyperinflammation, compensatory immunosuppression, endothelial barrier injury, or procoagulant activation, each with different implications for monitoring intensity and targeted perioperative management [176,177].

Biomarkers also enable a more precision-oriented perioperative approach. By identifying patient-specific dominant pathways, clinicians can better tailor priorities, for example, heightened diagnostic vigilance for infection in patients showing immune suppression, or closer haemodynamic/ventilatory and fluid stewardship when endothelial vulnerability is suggested [177,178,179].

Finally, biomarkers can be measured repeatedly, allowing for longitudinal risk assessment rather than a single static estimate. In many contexts, trends (kinetics) provide more actionable information than isolated values, and perioperative biomarker trajectories have been investigated specifically as a way to improve early identification of patients at risk of postoperative complications, including cardiovascular events or PPCs [178,180,181,182,183,184]. Overall, integrating biomarkers into perioperative care is not merely additive to existing prediction tools; it shifts PPC risk assessment toward a dynamic, biology-informed framework that is better aligned with the evolving nature of postoperative pulmonary pathophysiology [47,173,177].

## 6. Biomarkers and Risk Prediction in PPCs

Blood biomarkers have emerged as powerful tools for understanding the complex biological landscape that predisposes surgical patients to PPCs [47,184].

Rather than inferring vulnerability from observable characteristics alone, biomarkers reveal inflammatory activation, immune suppression, endothelial compromise, oxidative injury, coagulation abnormalities, and metabolic strain, processes that can precede clinically apparent PPCs [97,117,129,176,185,186]. Because these pathways evolve dynamically across the preoperative, intraoperative, and postoperative phases, the most informative approach is to interpret biomarkers both by biological domain and by their perioperative trajectory, preceding pulmonary decline [184,187]. Therefore, the true value of any biomarker rests not only on its biological plausibility but on the empirical evidence demonstrating its relevance to clinical outcomes [47,173].

Over the past two decades, numerous observational studies, mechanistic investigations, and translational research efforts have sought to determine whether specific biomarkers can reliably predict or detect PPCs [47,173,184]. A summary of clinical biomarkers relevant to perioperative inflammation, infection and pulmonary complications is shown in Table 1.

### 6.1. Inflammatory Biomarkers

Inflammatory biomarkers represent the most intuitive and historically recognized category of predictors for postoperative complications. Surgery induces a controlled form of trauma, and the body’s response to this trauma hinges on inflammatory mediator release. The magnitude and timing of this response vary widely among patients; inflammatory biomarkers aim to capture this variability, thereby flagging patients whose response is exaggerated, prolonged, or complicated by early infection, states that can amplify pulmonary vascular permeability, promote oedema, and facilitate neutrophilic lung injury [12,91].

At the heart of the inflammatory response lies CRP, a hepatically synthesized molecule whose plasma concentration can rise dramatically (often by orders of magnitude) within 24–48 h of a major inflammatory stimulus [188,189]. Traditionally regarded as a downstream marker of inflammation, CRP also participates in innate immune defence. It binds to phosphocholine and other ligands exposed on damaged or stressed cells and microbial surfaces, can trigger classical complement activation via C1, and enhances opsonization and clearance by phagocytes, features that make CRP both a mediator and a marker of inflammation [190,191,192].

Evidence patterns support CRP’s value at multiple time points. Preoperative CRP, for example, has been associated with increased postoperative morbidity in several surgical settings, consistent with the concept that a pre-existing inflammatory burden (i.e., chronic infection, metabolic disease, occult systemic stress) can prime patients for worse postoperative trajectories [193,194]. These insights emphasize that, for some patients, clinically relevant inflammation does not begin during surgery; rather, it precedes it and may set the stage for postoperative deterioration [193,194].

Postoperatively, CRP rises in a relatively predictable kinetic pattern (often peaking around postoperative day 2–3 after uncomplicated surgery), but early, exaggerated, or sustained CRP elevations can suggest complications rather than “normal” surgical inflammation [189,195]. In particular, higher CRP levels later in the early postoperative course have been observed in patients who develop postoperative infections such as pneumonia, supporting CRP as a useful adjunct signal when evaluating respiratory decline in the postoperative period [195,196].

PCT, although often classified primarily as an infection biomarker, also plays an important role in predicting and characterizing PPCs. PCT is particularly responsive to bacterial infection and systemic bacterial burden, yet it can also rise after major surgery as part of the systemic stress response [197,198,199]. In contrast to CRP, PCT generally rises earlier after a triggering stimulus and tends to decline more rapidly as the driver resolves, making it a dynamic marker of evolving systemic stress rather than a purely static indicator [197,198]. In postoperative patients, a progressive or disproportionate rise in PCT may indicate developing bacterial pneumonia or another infectious complication before radiographic findings or definitive bedside clinical signs become evident; conversely, stable or falling PCT values may support a non-bacterial explanation for respiratory symptoms [196,200]. Therefore, PCT can refine the differential diagnosis of PPCs by orienting clinical reasoning toward (or away from) bacterial aetiology, supporting more timely and targeted antimicrobial decisions while still requiring integration with microbiology (culture and/or molecular testing of respiratory specimens) for organism confirmation and definitive management [198,200].

Interleukins offer earlier, upstream insight into inflammatory activation. IL-6, a key cytokine during surgical stress, helps orchestrate the acute-phase response and is a principal inducer of acute-phase protein synthesis, including CRP [91,201]. Higher perioperative IL-6 levels and specific perioperative cytokine patterns have been associated with increased risk of postoperative complications in surgical cohorts, including thoracic surgery populations in whom PPCs are common [90,202,203]. IL-8 provides complementary information as a potent neutrophil chemoattractant/activator and has been strongly implicated in lung inflammation and injury syndromes characterized by neutrophilic infiltration [204]. IL-10, in contrast, reflects counter-regulatory anti-inflammatory signalling; perioperative increases in IL-10 have been linked to postoperative immune dysfunction (including impaired monocyte function), which is mechanistically relevant to susceptibility to postoperative infections such as pneumonia [97,106,205]. Taken together, interleukin patterns function as molecular fingerprints of how an individual patient’s immune system is reacting to surgery [97,106,203].

Finally, TNF-α, though often transiently elevated, can exert outsized effects on vascular endothelium and capillary leak, including in the pulmonary circulation [76]. Perioperative TNF-α (together with IL-6/IL-8) has been examined in relation to PPCs in operative settings, supporting a link between exaggerated cytokine release and pulmonary complications [93,202].

### 6.2. Immune Cell-Derived Biomarkers

A striking feature of the postoperative period is the coexistence of inflammation and immune suppression. Immediately after surgical trauma, the innate immune system becomes hyperactivated, leading to cytokine release and acute inflammation; this is then followed by a counter-regulatory anti-inflammatory phase that may overshoot, culminating in postoperative immunosuppression or even immune paralysis. Immune cell-derived biomarkers provide a practical window into this balance and are particularly relevant for understanding and anticipating infection-driven PPCs [22,94,107,108].

Among these biomarkers, the NLR has gained popularity because it is rapidly available and easy to interpret. Derived from a standard complete blood count, NLR reflects the interplay between innate activation (neutrophilia) and adaptive suppression (relative lymphopenia). Elevated NLR values preoperatively or postoperatively therefore suggest a biological milieu characterized by heightened inflammation and weakened immune surveillance. Across multiple surgical settings, including thoracic oncologic surgery, upper gastrointestinal cancer surgery, and esophagectomy cohorts, higher NLR has been associated with increased rates of postoperative pulmonary complications and/or postoperative pneumonia, supporting its role as a simple marker to flag patients who may benefit from closer respiratory and infectious surveillance [206,207,208,209].

Lymphopenia itself carries deep biological significance. Surgical stress responses (including neuroendocrine activation) can drive lymphocyte redistribution and apoptosis, compromising antimicrobial defence and impairing both viral and bacterial clearance. Clinically, persistent postoperative lymphopenia has been linked to infectious complications, including postoperative pneumonia after lung cancer surgery, underscoring that lymphocyte trajectories can reflect a system drifting toward immune exhaustion rather than recovery [95,96,107].

Monocyte-related biomarkers add another layer of insight. Monocyte distribution width (MDW), an “extended complete blood count” parameter, reflects morphologic variability in circulating monocytes during immune activation and has been studied as an early marker of sepsis and serious postoperative infection; in perioperative patients, rising MDW may therefore help raise early suspicion for evolving bacterial infection (including pneumonia when clinically compatible) [210,211,212,213]. Even more specific is monocyte HLA-DR expression: HLA-DR reflects monocyte antigen-presenting capacity, and sustained postoperative reductions are a well-described feature of postoperative immune suppression and have been associated with subsequent infection risk, including postoperative pneumonia in cardiothoracic settings [97,98,102,214].

Finally, immature granulocytes (IGs), released during bone marrow “stress” myelopoiesis, further illuminate innate immune dynamics. In surgical and critical care populations, early IG elevations can precede changes in total WBC count and have been investigated as early indicators of postoperative infection/sepsis, making them a potentially useful adjunct when interpreting early postoperative infectious risk (including infection-driven PPCs) [215,216,217,218].

### 6.3. Endothelial Injury Biomarkers

The pulmonary endothelium is a highly specialised and metabolically active interface that regulates vascular tone and permeability, maintains fluid balance, coordinates immune-cell trafficking, and contributes to coagulation within the alveolar-capillary unit [73,219]. In the perioperative setting, endothelial injury is increasingly recognised as an early and consequential driver of pulmonary dysfunction, and it can contribute to the progression from subclinical vascular leak to overt lung injury and ARDS [73,86]. Biomarkers of endothelial activation, such as soluble ICAM-1, soluble VCAM-1, von Willebrand factor (vWF), and angiopoietins, can reveal disruptions in vascular integrity and endothelial activation that may remain clinically silent during early bedside assessment [86]. Endothelial cells exposed to surgical stress, hypoxia, systemic cytokines, and mechanical ventilation upregulate adhesion pathways and may shed adhesion molecules into the circulation, reflecting enhanced leukocyte adhesion and transendothelial migration, core processes in inflammatory lung injury [110,220]. Consistent with this biology, higher circulating soluble ICAM-1 has been associated with worse outcomes in ARDS cohorts, supporting its role as a marker of clinically relevant endothelial injury [221], while soluble VCAM-1 is elevated in bronchoalveolar lavage fluid in ARDS, further linking endothelial activation to disease severity within the lung compartment [222]. vWF provides complementary information because it is stored in endothelial Weibel-Palade bodies and released during inflammatory and mechanical stress; elevated vWF antigen has repeatedly been associated with poorer outcomes in ARDS and in both septic and non-septic ARDS populations, consistent with diffuse endothelial activation and capillary leak physiology [112,113,223]. Angiopoietins add a mechanistically informative dimension: angiopoietin-1 supports vascular stability via tyrosine kinase-2 (Tie2) signalling, whereas angiopoietin-2 promotes endothelial destabilisation and permeability; accordingly, circulating angiopoietin-2 (and a higher angiopoietin-2/angiopoietin-1 ratio) correlates with lung injury severity and mortality in ARDS cohorts, including surgical ICU populations [224,225]. Importantly for perioperative medicine, angiopoietin dysregulation has also been documented after cardiopulmonary bypass, and higher postoperative angiopoietin-2 levels have been linked to endothelial hyperpermeability and to clinically meaningful complications, including postoperative respiratory failure, suggesting a potential window for earlier risk identification and more targeted fluid/ventilatory strategies in high-risk surgical patients [45,114,226,227].

### 6.4. Oxidative Stress Biomarkers

Oxidative stress contributes to PPCs by injuring alveolar epithelial and pulmonary endothelial cells, oxidising membrane lipids and proteins, impairing surfactant function, and amplifying inflammatory signalling within the lung [80,117]. In the perioperative setting, multiple exposures converge to increase reactive oxygen and nitrogen species generation, including systemic inflammation and catecholamine-driven metabolic stress, ischaemia-reperfusion events during surgical manipulation, exposure to supraphysiological inspired oxygen concentrations, and mechanical ventilation-related stretch/biophysical stress [80,117,118,228]. Importantly, reactive oxygen species can directly disrupt surfactant integrity, through oxidation of surfactant phospholipids and proteins, thereby worsening alveolar instability and promoting atelectasis and hypoxaemia, which can set the stage for downstream lung injury [229].

Among measurable biomarkers, malondialdehyde (MDA) is one of the most frequently reported indicators of lipid peroxidation and oxidative membrane damage. In thoracic surgery, intraoperative and perioperative MDA increases have been documented, particularly during lung re-expansion after one-lung ventilation, and higher oxidative stress has been associated with clinically relevant postoperative adverse events, including acute respiratory failure in higher-exposure groups [230]. Beyond the surgical setting, lipid peroxidation products (including MDA) are elevated in established ARDS, supporting the concept that oxidative membrane damage tracks with severe pulmonary dysfunction and poorer outcomes [231,232].

F2-isoprostanes (i.e., 8-iso-PGF2α/“8-isoprostane”) provide complementary information as highly specific products of free-radical-driven lipid peroxidation. Levels of isoprostanes measured in exhaled breath condensate are elevated in patients with ARDS, and circulating or exhaled isoprostane signals have been linked to outcome, reinforcing their value as markers of clinically meaningful pulmonary oxidative injury [233,234].

NO metabolites reflect another dimension of perioperative redox biology. NO is a key regulator of vascular tone and endothelial homeostasis with antiplatelet and anti-adhesive effects; however, under oxidative stress, NO can be consumed by reaction with superoxide to form peroxynitrite, a potent oxidant that exacerbates endothelial injury and microvascular dysfunction [235,236,237]. Perioperative studies have therefore incorporated NO alongside lipid peroxidation markers (including MDA) when quantifying hyperoxia-related redox perturbations during anaesthesia [228].

Finally, mitochondrial dysfunction, often driven or worsened by oxidative stress, has important implications for PPC risk because mitochondria are central to ATP production in both respiratory muscles and immune cells. Experimental and translational work shows that controlled mechanical ventilation can induce diaphragmatic mitochondrial oxidative damage and dysfunction (a key mechanism in ventilator-induced diaphragmatic dysfunction), which may reduce ventilatory reserve in the vulnerable postoperative period [125,238]. Although still emerging as perioperative tools, blood-based biomarkers of mitochondrial injury/dysfunction (notably circulating mitochondrial DNA) have been systematically linked to ARDS and are being explored for risk stratification in severe pulmonary injury states [127]. Clinically, oxidative stress signatures (including higher MDA and altered NO-related measures) have also been associated with difficulty liberating patients from mechanical ventilation, consistent with a link between systemic redox stress and functional respiratory compromise [239].

### 6.5. Coagulation and Fibrinolysis Biomarkers

Coagulation activation and PPCs are tightly interconnected through bidirectional “cross-talk” between inflammation and haemostasis. Pro-inflammatory mediators can upregulate tissue factor-driven thrombin generation while simultaneously downregulating endogenous anticoagulant and fibrinolytic pathways, creating a postoperative prothrombotic milieu [240,241]. Within the pulmonary circulation, this response can promote microvascular thrombosis and intrapulmonary coagulation abnormalities that contribute to ventilation-perfusion heterogeneity (increased dead space), impaired gas exchange, and worsening hypoxemia, mechanisms well described in ARDS pathobiology and relevant to severe PPCs [242,243].

Among measurable biomarkers, D-dimer (a fibrin degradation product) reflects activation of coagulation with secondary fibrinolysis, but it is also frequently elevated after surgery because it integrates thrombin formation, endothelial injury, and systemic inflammation [137]. In ARDS cohorts, higher circulating markers of coagulation and fibrinolysis, including D-dimer, have been associated with worse clinical outcomes, supporting a link between coagulation dysregulation and respiratory failure biology [244,245]. Clinically, persistently or disproportionately elevated postoperative D-dimer should therefore heighten vigilance for thrombotic PPCs (notably pulmonary embolism), while a low D-dimer, interpreted in the appropriate clinical probability context, retains value for ruling out pulmonary embolism in validated diagnostic strategies [246,247].

Fibrinogen, a classic acute-phase reactant, rises with inflammation and contributes to hypercoagulability; in the lung, disordered coagulation/fibrinolysis promotes fibrin deposition in airspaces and parenchyma, worsening oxygen diffusion and lung mechanics [248,249]. Finally, platelet activation amplifies immunothrombosis and microvascular injury via platelet-leukocyte endothelial interactions, with P-selectin being a key mediator in these processes [250,251]. Platelet activation indices such as mean platelet volume have been associated with thrombotic risk (including pulmonary embolism) and may capture a prothrombotic-proinflammatory platelet phenotype, although interpretability requires awareness of preanalytical and biological confounders [252,253].

### 6.6. Metabolic and Nutritional Biomarkers

The metabolic state of a patient undergoing surgery provides critical insight into physiological reserve, respiratory muscle endurance, and the ability to tolerate postoperative stress. Lactate is one of the most informative metabolic biomarkers because it reflects the balance between oxygen delivery and metabolic demand and is widely used as an integrative marker of global tissue stress in perioperative and critical care settings [163,254]. In major surgery cohorts, higher early postoperative lactate values and impaired lactate clearance have been consistently associated with increased postoperative morbidity and worse outcomes [255,256]. In patients with limited cardiopulmonary reserve, perioperative hyperlactatemia has also been linked to delayed extubation and prolonged mechanical ventilation, supporting its relevance to postoperative respiratory trajectories [257,258].

Albumin, although often framed as a nutritional marker, carries broader physiological meaning. Low albumin reflects a combination of chronic inflammation, reduced nutritional reserve, and increased capillary leak, and it is recognized as a relevant laboratory predictor of postoperative pulmonary risk in perioperative guidance [259]. In thoracic surgical populations, both preoperative hypoalbuminemia and perioperative albumin decline have been associated with a higher incidence of PPCs [165]. Mechanistically, hypoalbuminemia reduces plasma oncotic pressure and, especially when coupled with inflammation-driven endothelial leak, favours interstitial and alveolar fluid accumulation, contributing to pulmonary oedema and impaired gas exchange [260,261]. More broadly, hypoalbuminemia also tracks inflammatory burden and has been linked to impaired host defence and susceptibility to infectious complications, which is highly relevant to postoperative pneumonia risk [260,262].

Prealbumin (transthyretin), with its shorter half-life compared with albumin, provides a more dynamic signal of acute changes in nutritional and inflammatory stress [263,264]. In surgical cohorts (including pulmonary resection), lower preoperative prealbumin has been associated with postoperative morbidity and has been studied in relation to ventilatory support needs [265]. Taken together, lactate, albumin, and prealbumin offer complementary information on metabolic stress, vascular leak/inflammation, and physiological resilience, factors that jointly shape postoperative pulmonary vulnerability.

### 6.7. Emerging Omics-Based Biomarkers

Recent advances in genomics, transcriptomics, proteomics, and metabolomics are reshaping how perioperative biological risk is conceptualised, moving beyond clinical phenotypes toward molecularly defined vulnerability, although, at present, most omics approaches remain confined to research workflows rather than routine perioperative practice [266,267,268].

Genomic markers illustrate how inherited variation may predispose individuals to exaggerated inflammation, impaired epithelial-endothelial repair, or reduced pulmonary resilience. For example, variants in surfactant protein genes have been associated with susceptibility to severe lung injury and/or worse outcomes in ARDS-related cohorts [269,270,271,272]. Likewise, polymorphisms in endothelial and inflammatory regulators (i.e., VEGF and TNF-pathway-related variants) have been linked to ARDS prognosis and inter-individual differences in host response [268,273,274].

At the transcriptomic level, circulating microRNAs have gained attention because they can shift rapidly in response to systemic stress and modulate immune and inflammatory signalling. MicroRNAs such as miR-155 and miR-146a regulate innate immune pathways and immune-cell behaviour, and altered circulating levels have been investigated as early diagnostic/prognostic signals in acute lung injury and sepsis-associated lung injury, settings that share mechanistic overlap with severe PPCs trajectories [275,276,277,278].

Proteomic signatures offer a multidimensional view of interacting pathways (inflammation, coagulation, endothelial injury, metabolic stress), and proteomics is increasingly used to identify disease subphenotypes and candidate biomarker panels relevant to acute lung injury biology [266,267,279]. Metabolomics, meanwhile, captures downstream shifts in energy utilisation, lipid handling, and redox balance, providing a systems-level readout of physiological stress that may complement inflammatory markers and help refine phenotyping and prognosis in lung injury states [280].

Collectively, these omics technologies emphasise that PPCs and perioperative respiratory deterioration are rarely driven by isolated molecular events; rather, they emerge from integrated, system-wide biological patterns. Although not yet deployed broadly in clinical perioperative pathways, omics-derived biomarkers represent a key frontier for precision perioperative medicine, with potential to improve prediction and to uncover actionable therapeutic targets [266,267,268].

## 7. The Integration of Biomarkers into Predictive Models and Artificial Intelligence Systems

As perioperative medicine moves toward precision-oriented strategies, predictive models increasingly aim to combine clinical variables with perioperative biomarker signals to improve early identification of patients at risk for PPCs and postoperative pulmonary infection, including machine learning (ML), neural networks, and modern statistical modelling, to generate more individualized estimates of PPC risk [281,282]. Many studies report good predictive performance (i.e., discrimination measured by AUROC, often ~0.80 and in some cohorts > 0.85–0.90), particularly when biomarker kinetics are integrated with clinical features [283,284,285].

However, strong predictive performance does not necessarily translate into *clinical utility*. Utility depends on whether a model changes management in a way that improves patient-relevant outcomes (i.e., fewer severe PPCs, shorter length of stay) without undue harms such as unnecessary investigations or antibiotics. Accordingly, biomarker-augmented models should be evaluated not only for discrimination and calibration, but also for actionability (clear thresholds, workflow integration) and prospective impact. For example, multicentre ML models have demonstrated higher AUC than established clinical scores such as ARISCAT and LAS VEGAS in predicting PPCs [284]. Similarly, thoracic-surgery ML frameworks have identified laboratory features, including CRP, as key contributors to PPC risk, supporting the concept that biomarkers add a mechanistic layer to phenotypic risk assessment [283,286]. Beyond CRP, immune cell-derived markers such as the NLR have been associated with PPC risk in lung-cancer surgical cohorts [105], while infection-oriented biomarkers such as PCT, alone or combined with cytokine dynamics, can aid early identification of postoperative pneumonia in selected settings [44,287].

A further advantage of AI-enabled modelling is its ability to capture nonlinear interactions among inflammatory, immune, endothelial, and metabolic signals, relationships that are difficult to detect with conventional regression alone, enabling risk estimates to evolve as perioperative laboratory values and physiology change (“dynamic prediction”) [281,282]. At the same time, current AI-enabled models have important limitations, including cohort dependency and limited transportability across institutions, risk of overfitting (especially with high-dimensional biomarker panels), missing-data and measurement-heterogeneity challenges, and the need for robust external and prospective validation before routine clinical deployment [284,285,286]. Ultimately, the goal is not prediction alone but clinically actionable decision support, linking dynamic risk estimates to tailored postoperative monitoring, diagnostic escalation, and targeted preventive or therapeutic interventions.

## 8. Limitations of Current Research and Future Directions for Perioperative Precision Medicine

While biomarkers have demonstrated substantial potential to improve postoperative pulmonary risk assessment, their translation into routine perioperative workflows remains limited by scientific, methodological, logistical, and conceptual barriers [12,173,288]. Understanding these constraints is essential for interpreting the evidence responsibly and for designing clinically usable biomarker-driven strategies.

Despite extensive research, biomarker use in predicting and managing PPCs is still far from standardized, and a key obstacle is biological variability. Inflammatory mediators, immune-cell distributions, and endothelial activation markers fluctuate not only in response to surgical injury but also due to comorbidities, medications (i.e., steroids), baseline immune state, and circadian dynamics, factors that complicate clinical interpretation and undermine the reliability of single thresholds [106,288]. This variability makes it difficult to define universally valid cut-offs; the same CRP elevation, for example, can reflect chronic inflammatory disease rather than perioperative deterioration, while lymphocyte patterns may be altered by stress neuroendocrine responses or pharmacologic effects [106].

A second limitation concerns timing and biomarker kinetics. Biomarkers rise and fall on different trajectories (i.e., early cytokine changes versus later acute-phase responses), and their clinical meaning depends heavily on when they are sampled. Studies often use heterogeneous sampling windows (pre-op, end of surgery, post-operatory day 1 to 3, etc.), which makes cross-study comparisons challenging and weakens generalisability; importantly, kinetic/trajectory information may outperform single time-point measurements [184]. Without standardised measurement windows and trajectory-aware interpretation, biomarker-informed decisions risk inconsistency across institutions and surgical contexts [184,288].

Specificity is another major challenge. Many biomarkers implicated in PPCs also rise in non-pulmonary postoperative pathology (anastomotic leak, ileus, myocardial injury, systemic infection), blurring the distinction between pulmonary-specific complications and systemic inflammatory/infectious processes. This matters because PPCs themselves comprise a heterogeneous set of syndromes, pneumonia, atelectasis, aspiration, bronchospasm, pulmonary oedema, and ARDS, each with distinct mechanisms and biomarker relevance [12]. Overgeneralising a biomarker signal across PPC subtypes risks misclassification and inappropriate interventions [12,288].

Methodologically, the field is also constrained by variability in study design: small single-centre cohorts, heterogeneous procedures and perioperative pathways, inconsistent PPC definitions, and limited longitudinal sampling. These issues limit reproducibility and slow clinical adoption, mirroring broader concerns about the clinical utility and validation of many perioperative prediction tools [173,289]. Addressing these gaps requires large, prospective, multicentre studies with harmonised protocols, repeated sampling, and rigorous clinical adjudication of pulmonary outcomes [173,289].

Technological and logistical barriers further impede implementation. Many higher-resolution biomarkers (selected cytokines, endothelial markers, and omics-derived signatures) require specialised assays, strict preanalytical handling, and laboratory infrastructure that are not universally available. Although point-of-care cytokine testing is advancing, translating it into robust perioperative monitoring still requires validated devices, workflow integration, and clear clinical action thresholds [290,291]. Recent work demonstrates the feasibility of rapid cytokine measurement platforms and microfluidic assays capable of detecting cytokines (and even combining cytokines with CRP) in short turnaround times, but these approaches remain largely outside routine perioperative care pathways [291,292,293,294].

Looking forward, several converging developments could accelerate clinically meaningful translation. First, standardised multimarker panels spanning complementary biological domains may outperform single biomarkers by capturing the convergent pathobiology that precedes PPCs (inflammation, immune dysregulation, endothelial injury, coagulation stress, and metabolic strain) while also improving robustness to inter-individual variability [106,288]. Second, embedding biomarkers into dynamic, validated predictive algorithms, with careful attention to calibration, transportability, and prospective evaluation, may allow risk estimates to update over time, rather than relying on static preoperative scoring [177,289].

Third, continued progress in measurement technology is likely to be pivotal. The direction of travel is toward rapid, multiplexed, low-volume testing (including microfluidics and other point-of-care test approaches) that can deliver actionable biomarker data within clinically relevant timeframes [290,291,292,294].

Finally, multi-omics approaches (genomics, transcriptomics, proteomics, metabolomics) and integrative analytics may help define biologically coherent perioperative phenotypes and uncover novel targets, but clinical translation will depend on reproducibility, standardisation, and pragmatic validation in real-world surgical populations [106,295,296,297,298,299,300].

## 9. Conclusions

This review adds value by organizing perioperative blood biomarkers for infectious and non-infectious PPCs into mechanistic domains and dynamic perioperative trajectories rather than simply enumerating candidate markers. Perioperative blood biomarkers provide a pragmatic window into the evolving host response that precedes many PPCs and may complement traditional clinical risk scores by capturing dynamic trajectories of inflammation, immune dysfunction, endothelial injury, oxidative stress, coagulation activation, and metabolic strain. From a practical standpoint, routinely available markers (CRP, PCT, leukocyte-derived indices, albumin, lactate) are the most immediately actionable when interpreted longitudinally to distinguish expected postoperative inflammation from evolving infection and to support early stratification. In contrast, most endothelial injury markers, specialized immune-function assays, and omics signatures remain largely investigational because of assay availability, biological variability, heterogeneous sampling windows, and the lack of standardized cut-offs and prospective outcome validation. Future progress will likely come from harmonized prospective studies using standardized PPC endpoints and from integrating multimarket kinetics into validated, transportable dynamic prediction tools that can be embedded as clinical decision support.

## Figures and Tables

**Table 1:** Summary of clinical biomarkers relevant to perioperative inflammation, infection and pulmonary complications.

Biomarker	Domain	Perioperative Kinetics	Clinical Utility	Key Limitations	Availability
CRP	Acute-phase inflammation	↑; peak 2–3 days after surgery	Trend; complication flag	Non-specific; any inflammation	Routine
PCT	Bacterial infection signal	Early ↑; faster ↓ than CRP	Bacterial infection support	Surgical stress ↑; non-specific	Routine (many)
IL-6	Early cytokine/stress	Hours ↑ (early)	Early risk signal	Timing/assay variability	Limited
IL-8	Neutrophil recruitment	Early ↑	Lung-injury phenotype	Non-specific; limited access	Limited
IL-10	Anti-inflammatory response	Early/variable ↑	Immune-dysregulation signal	Context-dependent	Limited
TNF-α	Pro-inflammatory mediator	Transient early ↑	Hyperinflammation context	Short half-life; non-specific	Limited
WBC	Global leukocyte response	Early shifts common	Screening adjunct	Steroids/stress dilute signal	Routine
NLR	Innate ↑/adaptive ↓ balance	Often ↑ pre/post-op	Risk flag; surveillance	Non-specific; steroids/stress	Routine (derived)
Lymphocyte count	Adaptive suppression	Post-op ↓; may persist	Infection susceptibility	Many confounders	Routine
MDW	Monocyte activation	Early ↑ (infection)	Early infection adjunct	Platform-dependent	Some labs
Immature granulocytes	Stress myelopoiesis	Early ↑	Infection adjunct	Non-specific	Many analyzers
mHLA-DR	Immune competence	↓ after major surgery	Immune suppression stratification	Flow/handling sensitive	Specialized
Albumin	Reserve + leak/inflammation	Often ↓ post-op	Baseline vulnerability	Fluid/hemodilution	Routine
Prealbumin	Nutrition/inflammation	More dynamic than albumin	Nutritional risk adjunct	Inflammation/renal factors	Routine (often)
Lactate	Global physiologic stress	Early ↑; clearance matters	Severity/perfusion marker	Non-specific; adrenergic meds	Routine
D-dimer	Thrombo-inflammation	Common post-op ↑	PE/DVT context aid	Very non-specific post-op	Routine
Fibrinogen	Acute-phase + coagulation	↑ with inflammation	Thrombo-inflammatory context	Acute-phase reactant	Routine
Platelet indices (MPV)	Platelet activation	Variable	Adjunct risk signal	Preanalytical variability	Routine (derived)
sICAM-1/sVCAM-1	Endothelial activation	↑ with barrier stress	Endothelial phenotype	Limited access; non-specific	Specialized
vWF	Endothelial injury	↑ with stress/injury	Endothelial injury context	Systemic confounding	Variable
Ang-2 (±ratio)	Permeability/Tie2	↑ with severe stress	Vascular leak risk	Assay/threshold gaps	Specialized
MDA	Oxidative stress	↑ with oxidative injury	Mechanistic phenotype	Not standardized; non-specific	Research/limited
F2-isoprostanes	Oxidative lipid injury	↑ in oxidative states	Higher-specificity oxidative stress signal	Specialized workflow	Research
NO metabolites	Redox/NO signaling	Context-dependent	Mechanistic signal	Measurement variability	Research
mtDNA	DAMP/mitochondrial injury	Emerging	Risk phenotyping	Not standardized	Research
miRNA/proteomic/metabolomic panels	Multi-domain signatures	Emerging	Future prediction panels	Validation/cost barriers	Research

CRP, C-reactive protein; PCT, procalcitonin; IL-6, interleukin-6; IL-8, interleukin-8; IL-10, interleukin-10; TNF-α, tumor necrosis factor-α; WBC, white blood cell; NLR, neutrophil-to-lymphocyte ratio; MDW, monocyte distribution width; PE, pulmonary embolism; DVT, deep vein thrombosis; MPV, mean platelet volume; vWF, von Willebrand factor; NO, nitric oxide; Ang-2, angiopoiteins-2; Tie2, tyrosine kinase-2; MDA, malondialdehyde; mtDNA, mitochondrial DNA; miRNA, microRNA.

## Data Availability

No new data were created or analyzed in this study.

## Supplementary Materials

The following supporting information can be downloaded at: https://www.mdpi.com/article/10.3390/jcm15020699/s1, Search strategy and Figure S1: Flow diagram.

## Abbreviations

The following abbreviations are used in this manuscript:

Ang-	Angiopoietins-
ARDS	Acute Respiratory Distress Syndrome
CARS	Compensatory Anti-Inflammatory Response
CRP	C-Reactive Protein
IG	Immature Granulocytes
IL-6	Interleukin-6
IL-8	Interleukin-8
IL-10	Interleukin-10
MDA	Malondialdehyde
miRNA	microRNA
ML	Machine Learning
MDR	Multidrug-Resistant
MDW	Monocyte Distribution Width
mtDNA	Mitochondrial DNA
NLR	Neutrophil-To-Lymphocyte Ratio
NO	Nitric Oxide
PCT	Procalcitonin
PPCs	Postoperative Pulmonary Complications
ROS	Reactive Oxygen Species
TAT	Thrombin-Antithrombin
Tie2	Tyrosine Kinase-2
TNF-α	Tumour Necrosis Factor-α
vWF	von Willebrand Factor
WBC	white blood cell
