# Supplementary material for: Perioperative Blood Biomarkers of Infectious and Non-Infectious Postoperative Pulmonary Complications: A Narrative Review
Source: J Clin Med. 2026 Jan 15;15(2):699. doi: 10.3390/jcm15020699 (PMC12841655; doi:10.3390/jcm15020699)

SUPPLEMENTAL MATERIALS

SEARCH STRATEGY

(“pulmonary complications” OR “postoperative pulmonary complications” OR “postoperative sepsis” OR “postoperative infection”) AND (“biomarkers” OR “inflammation mediators” OR “c-reactive protein” OR “procalcitonin” OR “inflammation” OR “interleukin” OR “cytokine” OR “tumor necrosis factor-alpha” OR “HLA-DR” OR “neutrophils” OR “lymphocytes” OR “malondialdehyde” OR “oxidative stress” OR “endothelial dysfunction”)

FIGURE S1. FLOW DIAGRAM.

Study flow diagram according to the Preferred Reporting Items for Systematic review and Meta-Analysis Protocols recommendations.

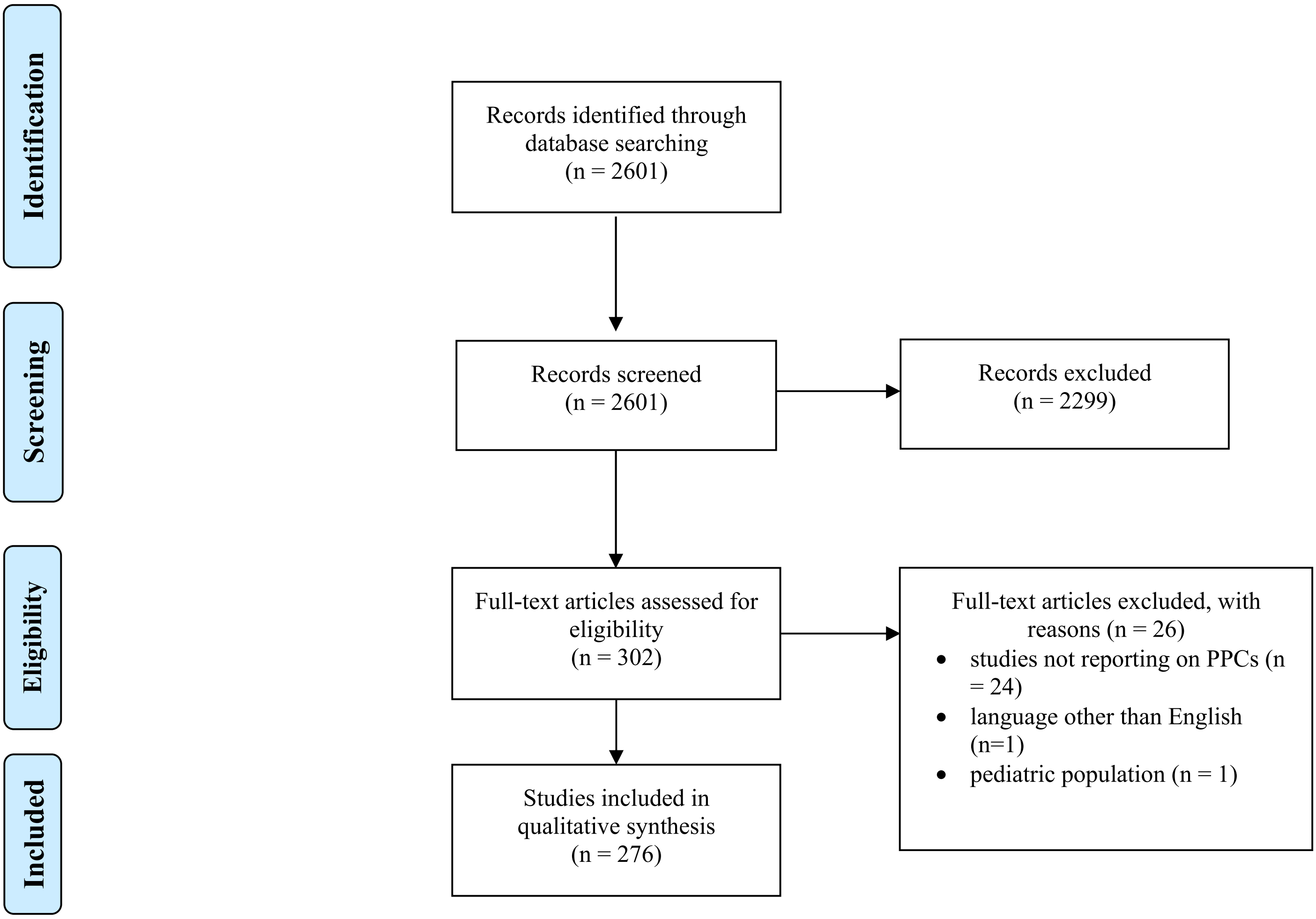

Supplement: Supplementary file 1 [file jcm-15-00699-s001.zip › jcm-4096363-supplementary.pdf]
